# Study protocol for the randomized controlled EVA (early vascular adjustments) trial: tailored treatment of mild hypertension in pregnancy to prevent severe hypertension and preeclampsia

**DOI:** 10.1186/s12884-020-03475-w

**Published:** 2020-12-12

**Authors:** Eva Mulder, Chahinda Ghossein-Doha, Evine Appelman, Sander van Kuijk, Luc Smits, Rogier van der Zanden, Joris van Drongelen, Marc Spaanderman

**Affiliations:** 1 Department of Obstetrics and Gynaecology, Maastricht University Medical Centre, PO box 5800, 6202 AZ Maastricht, the Netherlands; 2 Department of Obstetrics and Gynaecology, GROW - School for Oncology and Developmental Biology, Maastricht University Medical Centre, PO Box 616, 6200 MD Maastricht, the Netherlands; 3 Department of Cardiology, Maastricht University Medical Centre, PO box 5800, 6202 AZ Maastricht, the Netherlands; 4 Department of Clinical Epidemiology and Medical Technology Assessment (KEMTA), Maastricht University Medical Centre, PO box 5800, 6202 AZ Maastricht, the Netherlands; 5 Department of Epidemiology, Maastricht University, PO box 5800, 6202 AZ Maastricht, the Netherlands; 6 Department of Clinical Pharmacy and Toxicology/CARIM School for Cardiovascular Diseases, Maastricht University Medical Centre, PO box 5800, 6202 AZ Maastricht, the Netherlands; 7 Department of Obstetrics and Gynaecology, Radboud University Medical Centre, PO box 9101, GA Nijmegen, the Netherlands

**Keywords:** Gestational hypertension, Pregnancy, Preeclampsia, Haemodynamic profile, Tailored treatment, Antihypertensive drugs

## Abstract

**Background:**

In contrast to severe gestational hypertension, it is questioned whether antihypertensive medication for mild to moderate gestational hypertension prevents adverse maternal and offspring outcomes. Hypertensive drugs halve the risk of severe hypertension, but do not seem to prevent progression to preeclampsia or reduce the risk of complications in offspring. In fact, beta-blockers, a first line therapy option, are suspected to impair foetal growth. Disappointing effects of antihypertensive medication can be anticipated when the pharmacological mode of action does not match the underlying haemodynamic imbalance. Hypertension may result from 1) high cardiac output, low vascular resistance state, in which beta blockade is expected to be most effective, or 2) low cardiac output, high vascular resistance state where dihydropyridine calcium channel blockers or central-acting alpha agonists might be the best corrective medication. In the latter, beta-blockade might be maternally ineffective and even contribute to impaired foetal growth by keeping cardiac output low. We propose a randomized controlled trial to determine whether correcting the haemodynamic imbalance in women with mild to moderate hypertension reduces the development of severe hypertension and/or preeclampsia more than non-pharmacological treatment does, without alleged negative effects on foetal growth.

**Methods:**

Women diagnosed with mild to moderate hypertension without proteinuria or signs of other organ damage before 37 weeks of pregnancy are invited to participate in this randomized controlled trial. Women randomized to the intervention group will be prescribed tailored antihypertensive medication, using a simple diagnostic and treatment algorithm based on the mean arterial pressure/heart rate ratio, which serves as an easy-to-determine proxy for maternal circulatory state. Women randomized to the control group will receive non-pharmacological standard care according to national and international guidelines. In total, 208 women will be randomized in a 1:1 ratio. The primary outcome is progression to severe hypertension and preeclampsia and the secondary outcomes are adverse maternal and neonatal outcomes.

**Discussion:**

This trial will provide evidence of whether tailoring treatment of mild to moderate gestational hypertension to the individual haemodynamic profile prevents maternal disease progression.

**Trial registration:**

NCT02531490, registered on 24 August 2015.

## Background

Preeclampsia is a leading cause of maternal and perinatal morbidity and mortality in the western world [1]. In most cases of preeclampsia-related maternal death, blood pressure is not adequately controlled [2, 3]. For its well-established maternal benefits, it is generally accepted to treat blood pressure when it reaches the threshold of severe hypertension (systolic or diastolic blood pressure ≥ 160 or ≥ 110 mmHg respectively) [4]. In contrast, effectiveness of antihypertensive medication in mild to moderate hypertension is subject of debate and medication is not uniformly initiated because of different effects on maternal disease progression and alleged poorer foetal outcomes [5, 6]. In mild to moderate hypertension, the use of any antihypertensive drugs halves the risk of developing severe hypertension (relative risk of 0.49; 95% CI 0.40–0.60), but does not prevent progression to preeclampsia (relative risk of 0.93; 95% CI 0.80–1.08), or reduce the risk of offspring complications (relative risk for small for gestation age neonate, 0.97; 95% CI 0.80–1.17, relative risk for stillbirth, 1.14; 95% CI 0.60–2.17 and relative risk for neonatal death, 0.79; 95% CI 0.14–4.34) [6]. On the other hand, sub-grouped by class of drugs, beta-blockers seemed to reduce the development of preeclampsia (OR 0.73; 95%CI 0.57–0.94) but may increase the chance of the neonate being small for gestational age (OR 1.38; 95%CI 0.99–1.92), while calcium antagonists seemed to increase the chance of preeclampsia (OR 1.40; 95%CI 1.06–1.86), but have no effect on foetal growth (OR 0.84; 95%CI 0.60–1.16) [6]. As a consequence of these inconsistent findings when pooling all antihypertensives irrespective of mode of action, international guidelines recommend initiating medication of choice only in severe hypertension or repeated moderate gestational hypertension [7, 8].

A drawback in studies investigating treatment of mild to moderate gestational hypertension is that they do not consider the maternal circulatory state or the circulatory response to the blood pressure lowering drug. Instead, most reported treatments involve stepwise use of compound drug classes, based on the physician’s experience; in this treatment approach, chosen medications are only changed if severe side effects are observed or if increasing dosages do not achieve the target blood pressure. This trial-and-error approach may not only delay the time interval to effective circulatory control, but may also negatively affect foetal wellbeing that may prelude to preterm birth.

Antihypertensive drugs lower blood pressure via different mechanisms. Blood pressure is regulated within narrow boundaries by beat-to-beat baroreceptor-mediated alteration of cardiac output and vascular resistance. Blood pressure only changes when these two determinants are unable to compensate deteriorations; therefore, elevated blood pressure can be lowered by reducing cardiac output or vascular resistance, or both. Beta-blockers, central-acting alpha agonists, and calcium antagonists are commonly used to lower blood pressure during pregnancy. Selective beta-blockers lower elevated blood pressure predominantly by lowering the heart rate (HR) and reducing contractility, resulting in reduced cardiac output. Beta-blockers with additional ɑ-adrenergic receptor blockade activity, like labetalol, concurrently reduce peripheral resistance [9, 10]. Central-acting alpha receptor agonists, like methyldopa, lower blood pressure by altering central sympathetic activity. Cardiac output levels do not change, suggesting a balance between reduced afterload, venous return, and negative chronotropic and inotropic effects of sympathetic inhibition [11, 12]. Dihydropyridine calcium channel blockers, like nifedipine, are relatively vasoselective. They inhibit calcium ions from entering the calcium channels of vascular smooth muscle, so blood pressure is mainly lowered by reducing arterial tone and with it, peripheral vascular resistance. Secondary to vasodilation, the sympathetic nervous system is activated resulting in increased levels of norepinephrine, higher HR and ultimately increased cardiac output [13–15].

### Haemodynamic parameters in gestational hypertension

**Fig. 1:**
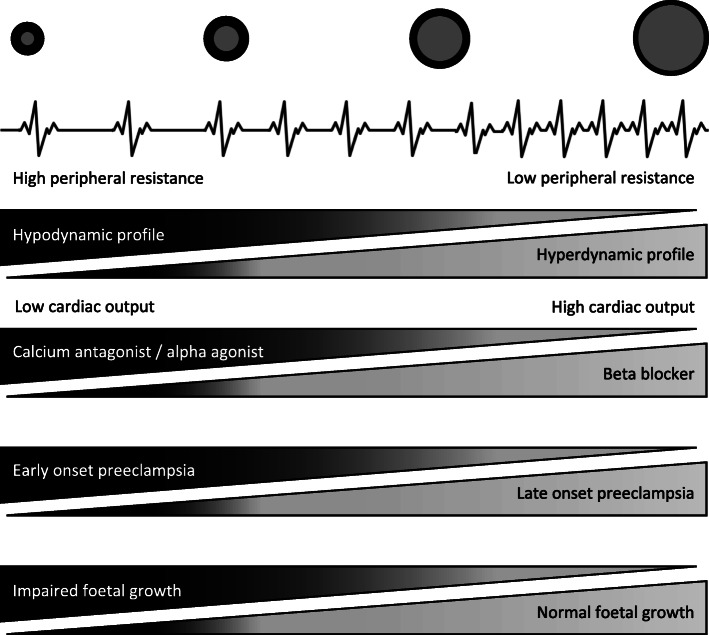
Haemodynamic profiles in mild hypertension, associated complications and appropriate antihypertensive treatment

The haemodynamic profile of gestational hypertension differs between individuals. On the one hand, gestational hypertension can originate from hyperdynamic circulation characterized by high cardiac output and low vascular resistance; this profile is often accompanied by late onset preeclampsia and normal foetal growth (Fig. 1). On the other hand, a hypertensive profile with high vascular resistance is associated with early onset maternal complications and impaired foetal growth [16–19]. In the clinical phase of preeclampsia, most women exhibit a high-resistance profile with either low cardiac output (in 58% of women) or normal cardiac output (in 36% of women) [20]. Altered pre-pregnant haemodynamic phenotypes, inadequate cardiovascular adaptation to pregnancy, or crossover from hyperdynamic circulation to the more unfavourable hypertensive circulation with high vascular resistance as a consequence of endothelial derangement and loss of intravascular fluid, might underlie the divergent haemodynamic profiles [21, 22]. These heterogeneous circulatory profiles may explain the variable results of trials on antihypertensive therapy in mild to moderate hypertension during pregnancy. Beta-blockers are thought to be suitable for treating hyperdynamic hypertension, but reducing cardiac output may be detrimental in hypertensive women with high vascular resistance who already have low-cardiac-output. These women may instead benefit from vasodilation by dihydropyridine calcium channel blockers, or from central-acting alpha agonists that reduce peripheral resistance.

### Tailored treatment of hypertension

In non-pregnant individuals with uncontrolled hypertension, haemodynamically tailored antihypertensive treatment almost doubled the chance of reaching the target blood pressure within 3 months of initiating or adjusting the therapy compared with conventional standard treatment based on the specialist’s preference [23, 24]. During pregnancy, with respect to underlying haemodynamic profile, adequate blood pressure response to beta-blockage can be predicted by the mean arterial pressure (MAP), HR, and (if available) stroke volume index [25]. About 20–25% of gestational hypertensive women were refractory to labetalol and needed additional vasodilatory therapy. In these women, cardiac output was lowest and vascular resistance was highest before treatment. The highest response rate to labetalol primarily relates to alteration of HR and peripheral vascular resistance, and consequently blood pressure, but not stroke volume. This suggests that a simple algorithm weighing blood pressure and HR may grossly indicate the underlying haemodynamic profile. Therefore, it may be better to view antihypertensive drugs as adjusters of the haemodynamic state rather than as reducers of blood pressure [26]. If we do not take the maternal haemodynamic profile and the pharmacological mode of action of the drug into account, generic antihypertensive treatment will continue to result in disappointing, ineffective or even paradoxical outcomes for at least part of treated mothers and their offspring. To address this problem, we propose a randomized trial to compare tailored antihypertensive treatment based on the maternal circulatory profile with generally practiced active surveillance of hypertension in pregnant women with mild to moderate hypertension.

## Methods/design

### Aim

The aim of this study is to determine whether haemodynamically tailored antihypertensive therapy in pregnant women with mild to moderate hypertension reduces the incidence of severe hypertension and preeclampsia and improves offspring outcome compared with standard care.

### Objectives

Our primary objective is to determine whether haemodynamically tailored treatment of mild to moderate hypertension reduces disease progression to severe hypertension and preeclampsia compared with standard care. Our secondary objectives are to assess the effects of this treatment approach on maternal and offspring outcomes, including concomitant HELLP syndrome and eclampsia, gestational age at delivery, and neonatal birth weight and centile.

### Trial design and setting

**Fig. 2:**
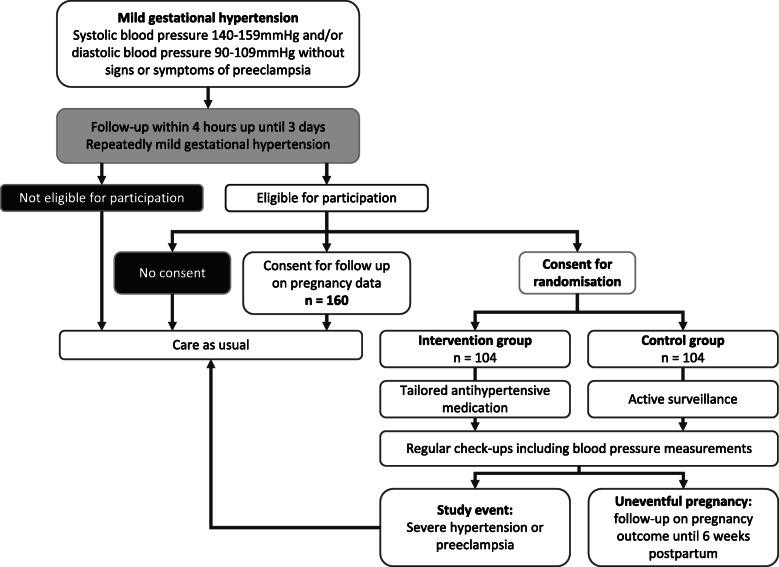
Schedule of enrolment, intervention, and assessments

**Fig. 3:**
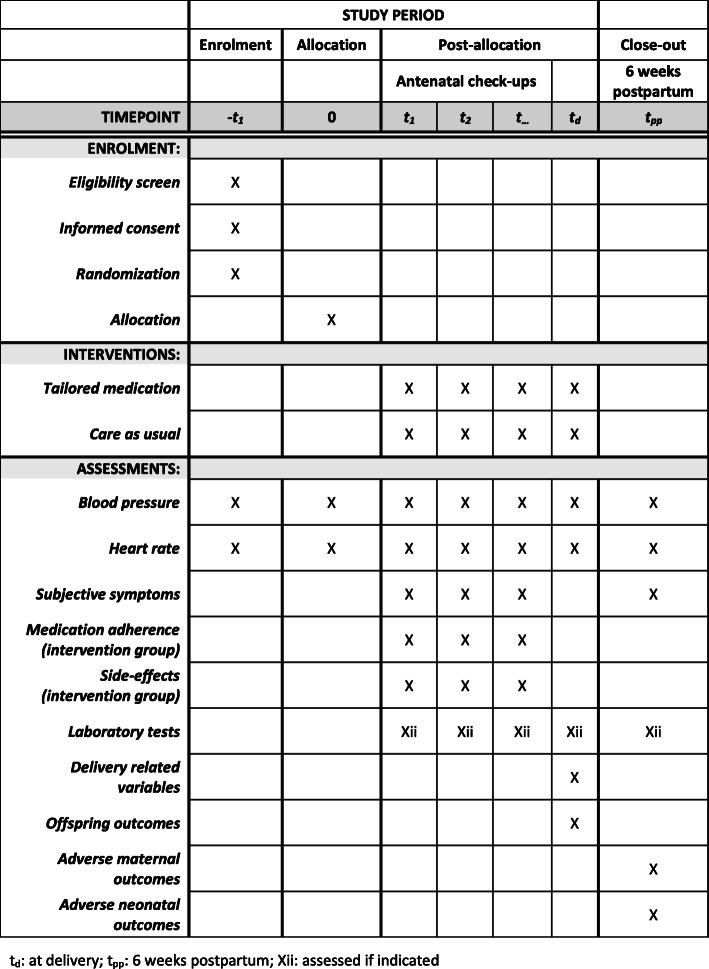
SPIRIT timetable of the study

The Early Vascular Adjustments (EVA) trial is a randomized controlled open-label superiority trial, with two parallel groups (intervention and control). Progression to severe hypertension or preeclampsia is the primary endpoint. A schematic overview of patient enrolment and follow-up, and the SPIRIT timetable for the study are presented in Figs. 2 and 3. Participants will be randomized 1:1 to the intervention or control group using premade, non-opaque sealed envelopes, in two blocks of 104 participants to achieve equal sample sizes in the end. Generalizability of the findings will be assessed in a third group of women who do not consent to randomization, but agree to follow-up of their pregnancy outcomes. In this group, women will receive standard care, meaning that their physician will discuss treatment with them, and decide which medication to prescribe.

Participants will be recruited from the outpatient clinic and department of obstetrics in Maastricht University Medical Centre, which is both a secondary and tertiary referring hospital. During every routine pregnancy check-up, blood pressure is measured. Women with mild to moderate hypertension (defined as systolic blood pressure measures ≥140 and < 160 mmHg and/or diastolic blood pressure ≥ 90 and < 110 mmHg) without symptoms or signs of preeclampsia will be given the study patient information form, and follow-up on blood pressure is planned at least 4 h later and within a few days. Eligible women who give consent will be randomized to the intervention or control group, after which they will continue regular pregnancy check-ups with blood pressure measurements; additional laboratory analysis will be performed as indicated. After 37 weeks’ gestation, induction of labour will be recommended to all participants, as this is associated with improved maternal outcome [27]. Study endpoints are an uneventful pregnancy and 6-week postpartum period, or diagnosis of severe hypertension or preeclampsia. In the latter case, participants will receive standard care, which usually means hospitalization and intensifying or initializing antihypertensive medication for the intervention group and control group, respectively.

### Eligibility criteria

Women must provide written informed consent before any study procedure occurs.

#### Inclusion criteria

Patients eligible for the trial must meet the following criteria at randomization:

- Age ≥ 18 years;
- Living foetus before 37 weeks’ gestation;
- Diagnosed with mild gestational hypertension (systolic blood pressure 140–159 mmHg and/or diastolic blood pressure 90–109 mmHg, measured twice with at least 4 h in between).

#### Exclusion criteria

- Severe hypertension (systolic blood pressure ≥ 160 mmHg and/or diastolic blood pressure ≥ 110 mmHg);
- Diagnosed with preeclampsia, HELLP syndrome, or eclampsia;
- Taking antihypertensive drugs;
- Intending to terminate the pregnancy;
- Foetus has a major anomaly or chromosomal abnormality;
- Not able to comprehend the study outline;
- Contraindication for one of the possible prescribed antihypertensive medications.

### Interventions

**Fig. 4:**
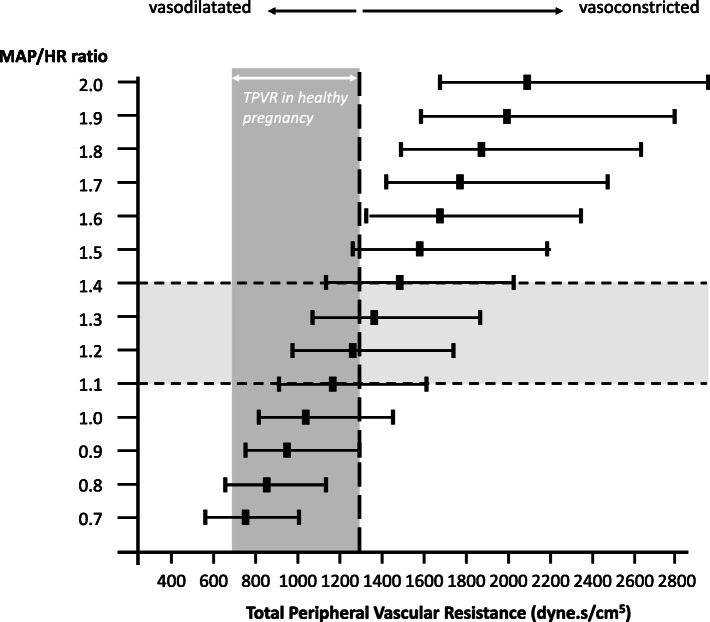
Assessment of underlying haemodynamic profile by MAP/HR ratio and the likelihood of vasoconstricted, low output or vasodilated high-output hypertension

**Fig. 5:**
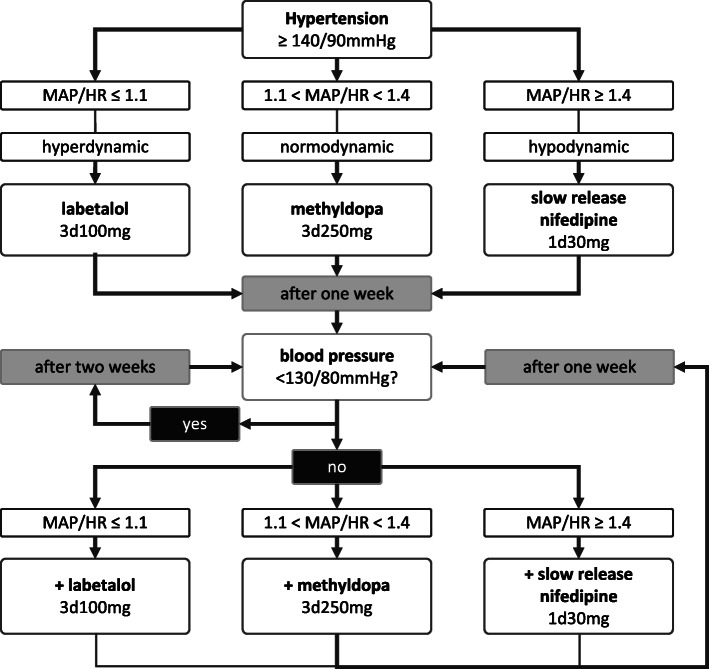
Tailored antihypertensive treatment strategy in the intervention group

Eligible patients who consent to participate will be randomized to receive tailored antihypertensive medication (intervention group) or standard care (control group). The antihypertensive medications we plan to administer to participants in the intervention group can be safely prescribed to pregnant women [6]. Total peripheral vascular resistance (calculated as: 80 × MAP / cardiac output) indicates whether the hypertension is cardiac- or vascular-driven. In a healthy pregnancy, total peripheral vascular resistance drops from approximately 1300 to 1000 dyne.s/cm^5^ during the second and third trimester [28, 29]. Considering a standard deviation (SD) of 150 dyne.s/cm^5^, the upper limit of healthy dilated total peripheral vascular resistance is 1300 dyne.s/cm^5^. Women can be considered vasoconstricted when gestational vascular resistance exceeds 1300 dyne.s/cm^5^ and vasodilated when vascular resistance is below 1300 dyne.s/cm^5^. To avoid the need for an additional tool to assess cardiac output (stroke volume × HR), the MAP/HR ratio is used as a proxy for the haemodynamic profile. The MAP/HR ratio differs from total peripheral vascular resistance assessment in that it does not account for stroke volume. During healthy pregnancy, the mean ± SD stroke volume is 75 ± 10 ml. In the estimation of which proportion of women can be assumed to be vasoconstricted or vasodilated using the MAP/HR ratio, we can estimate the range in which total peripheral vascular resistance must be by taking the mean ± 2 SD stroke volume. When the MAP/HR ratio exceeds 1.4, more than 95% of all women must be considered vasoconstrictive. Contrary, when the MAP/HR ratio is 1.1 or less, more than 50% of women are likely to have a vasodilated circulation (Fig. 4). In the absence of empirical data that support the determination of cut-off values, we assumed a likelihood of more than 95% vasoconstrictive and relatively hypodynamic, corresponding a MAP/HR ratio 1.4 as reference value for considering vasodilating medication, and opposite, a likelihood of more than 50% of women to be vasodilated and hyperdynamic, corresponding MAP/HR ratio of 1.1 as a reference value for considering HR-lowering and with it, cardiac output-lowering medication. In the latter case, a beta-blocker with dual alpha- and beta-adrenergic receptor antagonism will account for the potential overlap in profiles. Thus, we will administer labetalol in the intervention group when hyperdynamic hypertension is assumed (MAP/HR ratio ≤ 1.1), and slow-release nifedipine when hypodynamic hypertension is assumed (MAP/HR ratio ≥ 1.4). Women with normodynamic hypertension will be identified by a MAP/HR ratio between 1.1 and 1.4 and will be prescribed methyldopa (Fig. 5). Treatment will be increased if the targeted blood pressure of < 130/80 mmHg (MAP 97 mmHg) is not achieved [7, 30]. The maximum dosages for blood pressure control are 800 mg three times daily for labetalol, 30 mg three times daily for slow release nifedipine and 1000 mg three times daily for methyldopa. If the maximum dosage has been administered and the MAP/HR ratio does not indicate another medication class, the blood pressure will be accepted. Moreover, in the unlikely event that blood pressure measurements fall below 95/50 mmHg, the treatment regime will be reduced and the last added medication step will be stopped. In case of intolerable side effects, the medication class will be switched; methyldopa will substitute labetalol or nifedipine, and nifedipine will substitute methyldopa when the MAP/HR ratio is ≥1.3 and labetalol when the MAP/HR ratio is < 1.3. Antihypertensive drugs will be provided by the local pharmacy. Since this is an open-label study, the participant, researcher, and physician will know the type and dosage of the prescribed medication. Adherence to the treatment and side effects of the medication in the intervention group will be discussed and recorded during each scheduled check-up.

#### Example 1

A 34-year-old woman, eligible for participation is randomized for the intervention group. Her blood pressure is 145/80 (102) mmHg, with a HR of 60 bpm (MAP/HR ratio 1.7) and therefore she is prescribed slow release nifedipine 30 mg once a day, and at follow-up her blood pressure is 135/74 (94) mmHg, HR of 72 bpm (MAP/HR ratio 1.3). Therefore, methyldopa 250 mg 3 times daily is added. One week later her blood pressure is 118/74 (89) mmHg, HR of 74 bpm (MAP/HR ratio 1.2), which is below (additional) treatment threshold, and adequate blood pressure control is reached. Check-ups are continued every 2 weeks.

#### Example 2

A 23-year-old woman eligible for participation is randomized for the intervention group. Her blood pressure is 155/91 (112) mmHg, and her HR is 94 bpm (MAP/HR ratio 1.2). She is prescribed methyldopa 250 mg three times daily. At the follow-up visit, her blood pressure measures 141/85 (104) mmHg, HR is 101 bpm (MAP/HR ratio 1.0). As she did not achieve target blood pressure, labetalol 100 mg 3 times daily is added. One week later, blood pressure is 125/75 (92) mmHg, 92 bpm (MAP/HR ratio 1.0) and adequate blood pressure control is reached. Check-up are continued every 2 weeks.

### Outcome measures

#### Primary study endpoints

1. Severe hypertension, defined as a systolic blood pressure ≥ 160 mmHg and/or a diastolic blood pressure ≥ 110 mmHg [31].
2. Preeclampsia defined as hypertension and one or more of the following new-onset conditions:
  Proteinuria (spot urine protein/creatinine ≥30 g/mol or ≥ 300 mg/24 h);
  Renal insufficiency (creatinine levels ≥90 μmol/L);
  Liver involvement (elevated transaminases at least twice the upper limit of normal (≥70 U/I);
  Neurological complications (hyperreflexia when accompanied by clonus and/or severe headaches, persistent visual scotomata, altered mental status, eclampsia);
  Haematological complications (thrombocytopenia defined as platelet count below 150*10^9^/L, disseminated intravascular coagulation, haemolysis) [31].

#### Secondary and other study parameters

Maternal outcomes: side effects of medication, required level of care, serious maternal complications (maternal death, HELLP syndrome defined as the combination of thrombocytopenia, elevated transaminases and haemolysis, stroke, blindness, uncontrolled hypertension, respiratory failure, myocardial ischaemia/infarction, renal failure, hepatic haematoma and/or rupture, coagulopathy), birth-related variables, and placental abruption.

Neonatal outcomes: miscarriage, gestational age at delivery, birth weight and centile, Apgar score at 1 and 5 min after birth, use of antenatal corticosteroids and surfactant, adverse perinatal outcomes (miscarriage, stillbirth, neonatal mortality, and serious morbidity including neonatal sepsis, central nervous system morbidity and necrotizing enterocolitis), required level of care, and duration of care.

### Data collection

Data will be recorded in predesigned case record forms. Data on pregnancy and neonatal outcomes will be collected from the hospital maternity records. Additional neonatal outcomes will be collected from the discharge summary when neonates are admitted to the neonatal care department.

### Sample size / power calculation

Severe hypertension and preeclampsia develop respectively in 20% and 15–25% of women initially diagnosed with mild to moderate gestational hypertension [6, 32]. As severe hypertension and preeclampsia require comparable in-hospital treatment, we consider an 15% reduced incidence of both as clinically relevant. We calculated the sample size based on a progression level of 10% in the intervention group and a progression level of 25% in the control group. For a desired power of 80% and a two-tailed alpha level of 0.05, we need to recruit 99 women per group. Considering an anticipated dropout rate of 5%, we will recruit 208 women for randomization. We expect to include approximately 160 women who do not consent for randomization but agree to follow-up of their pregnancy outcomes. In this observational cohort, we will evaluate general obstetrical care for mild to moderate hypertension, patient characteristics, and patient outcomes comparable to the randomized population.

### Planned statistical analysis

The effectiveness of haemodynamically tailored treatment of mild to moderate gestational hypertension will be evaluated based on the intention-to-treat principle. Descriptive statistics will be used to compare baseline characteristics between study groups. The primary analysis will be an X^2^ test, or Fisher’s exact test in groups with less than five cases, to compare the incidence of severe hypertension and preeclampsia in the intervention group and the control group. We planned to conduct a per protocol sub-analysis in with women with at least 80% of their medication intake assessed by self-report at each pregnancy check-up. Secondary outcomes will be assessed by using X,^2^ Fisher’s exact test, Mann-Whitney U or T test as appropriate. For both primary and secondary outcomes, crude and adjusted odds ratio’s will be calculated (control group will be considerate as reference) and adjustments will be made for gestational age at recruitment. Separate analyses will be conducted to evaluate whether or not optimal blood pressure control was reached, if the type of medication and dosage affected the outcomes, which haemodynamic profiles were most prevalent, and if the initial haemodynamic profile affected the outcome, and to explore the effect of BMI, parity and age on the haemodynamic profile and response to medication.

## Discussion

Mild to moderate hypertension is a common complication during pregnancy, leading to increased maternal and foetal mortality and morbidity when it progresses to severe hypertension, preeclampsia and associated sequela. Previous studies show paradoxical maternal and foetal outcomes when a general, stepwise antihypertensive medication strategy is used. Previous studies on treating gestational hypertension have not considered the maternal circulatory profile and pharmaceutical mode of action of the prescribed medication, and the contemplated response to the medication class, while hypertensive pregnant women exhibit distinct circulatory profiles. This trial will investigate whether haemodynamically tailored treatment of mild to moderate gestational hypertension prevents progression to severe hypertension and preeclampsia without paradoxical maternal and offspring outcomes as observed in traditional non-tailored stepwise treatment approaches. We will tailor antihypertensive treatment to participants’ individual haemodynamic profiles using a simple diagnostic and treatment algorithm that includes the MAP and HR values. Directly measuring cardiac function might determine the underlying haemodynamic profile more precisely, but these methods (such as echocardiography) are labour-intensive and require expertise. Our designed algorithm represents a pragmatic approach to choosing appropriate antihypertensive drugs and can be easily implemented since both parameters are readily available in daily clinical practice.

## Data Availability

Not applicable.

## Abbreviations

EVA: Early Vascular Adjustments
HELLP: Haemolysis, elevated liver enzymes and a low platelet count
MAP: Mean arterial pressure
HR: Heart rate
